# The relevance of nitrogen-based, high-enthalpy plasmas for effective feedstock treatment in thermal spraying of suspensions

**DOI:** 10.1038/s41598-025-31662-2

**Published:** 2025-12-09

**Authors:** Georg Mauer, Robert Vaßen

**Affiliations:** 1 Institute of Energy Materials and Devices, IMD-2: Materials Synthesis and Processing, Forschungszentrum Jülich GmbH, Jülich, Germany; 2 Department of Mechanical Engineering, TU Dortmund University, Dortmund, Germany; 3 Department of Mechanical Engineering, Institute for Materials, Ruhr University Bochum, Bochum, Germany

**Keywords:** Plasma spraying, Plasma torch, Nitrogen, Enthalpy, Transport coefficient, Chemistry, Energy science and technology, Engineering, Materials science, Physics

## Abstract

This work aims to contribute to our understanding of the importance of nitrogen in enhancing feedstock treatment in plasma spraying. Three different plasma gas compositions—a ternary argon-based composition, a ternary nitrogen-based composition, and a nitrogen-based composition without argon—were used with the Axial III™ plasma torch for suspension plasma spraying. The thermodynamic and transport properties of the plasma gas mixtures were calculated and compared. Based on these calculations, the Ability of Acceleration Factors (AAF) and the Ability of Heating Factors (AHF) were determined and correlated with the measured in-flight particle velocities and temperatures. Computational and experimental results indicate that transport properties, specifically thermal conductivity and viscosity, are key factors in thermal feedstock treatment. In this respect, a high nitrogen content is advantageous. Therefore, nitrogen can be considered a high-heat-transfer component of the plasma. Nitrogen also introduces a relatively high enthalpy. Enthalpy describes the amount of energy stored in the plasma gas that can be released. If the energy output is not enough to heat the feedstock adequately, especially when dealing with high feed rates of liquid feedstocks, the enthalpy of the plasma gas can become a limiting factor. In such cases, a high nitrogen content in the plasma gas is advantageous because the higher enthalpy provides more energy than an argon-based plasma. However, this requires a thermally resilient torch concept.

## Introduction

Plasma spraying is a highly versatile coating processes^1^ that belongs to the broad category of thermal spray technology. It can process a wide variety of coating and substrate materials. The feedstocks are powders that are injected into a hot, fast jet generated by a plasma torch. If very fine, non-flowable powders are to be sprayed, a suspension can be used^2^.

The thermal spray community often talks about high enthalpy plasma torches. High plasma enthalpies are commonly considered to be beneficial for a high ability to heat and accelerate the feedstock, thereby increasing the productivity and efficiency of the spray process.

### Basics of enthalpy

The first thing to remember is the meaning of enthalpy. The enthalpy of a thermodynamic system is the sum of the system’s internal energy and its work of volume, which is the product of the system’s pressure and volume. The pressure–volume term expresses the work done against a constant external pressure to increase the volume of the system. The pressure–volume term is very small for solids or liquids and quite small for gases. Therefore, enthalpy is a proxy for energy in thermodynamic systems.

Enthalpies are usually given as specific values, i. e. either in terms of mass, volume or moles. Many thermal spray publications give mass-specific enthalpies *h*, e.g. in^1^, mostly expressed in MJ/kg. Among the commonly used plasma gases, hydrogen has the highest mass-specific enthalpy and argon has the lowest. When the enthalpy and mass density are multiplied, *h’* = *h* ∙ *ρ*, the mass dependence of each quantity is eliminated and the volumetric enthalpy is obtained. Taking *h’* into account, the differences between the gases are smaller. It is largest for nitrogen and hydrogen, while it is almost identical for argon and helium up to about 10,000K^3,4^.

Although thermodynamic functions and specific heats are commonly expressed in terms of mass, this can be misleading in the context of plasma spraying, where the plasma gas composition is usually expressed volumetrically. Therefore, volume flows at standard conditions are set at the control unit, usually expressed in standard liters per minute (slpm). For this reason, some authors have preferred to use volumetric enthalpies as these values are more convenient for practical interpretation^5,6^. They are also used in this paper. If the reference value is the volume at standard temperature and pressure (STP) 273.15 K and 100 kPa^7^, it must be noted that the density in the expression *h’* = *h* ∙ *ρ* is at STP and not at actual conditions, e.g. in the torch or in the plasma jet. In this case, STP conditions are indicated by the subscript “0” and *h*_*0*_*’* is usually expressed in kJ/sl (sl means standard liter). In contrast, *h’* is generally given in MJ/m^3^.

There are not only practical reasons for using volumetric enthalpies, but also mechanistic ones. If the effect of the proportion of a particular plasma gas on the transfer of energy from the arc to the plasma gas and on to the components of the torch is to be studied, the volumetric enthalpy of the gas is more meaningful than the mass-related enthalpy. This is because the energy transfer depends on the size of the surface area for these interactions occupied by that particular plasma gas. The size depends on its volumetric fraction in the total plasma gas mixture. This also argues in favor of using volumetric enthalpies.

Enthalpy values cannot be expressed as absolute values, but as enthalpy differences relative to a zero value, such as at room temperature. Since enthalpies cannot be measured directly, they are determined in plasma spray systems by measuring the temperature difference of the torch cooling water between the supply and return. Based on the water flow and the heat capacity of the water, the dissipated energy is calculated and subtracted from the total electrical input power *P*_*in*_ to obtain the net power *P*_*net*_. If thermal losses, e.g. by radiation, are neglected, it can be assumed that *P*_*net*_ is completely transferred to the plasma gas flow in the torch and corresponds to the enthalpy uptake per unit time. The specific plasma enthalpies *h* or *h’* are then obtained from the ratio of this uptake to the plasma gas mass or volume flow, respectively. If the volume flow at STP conditions is used, *h*_*0*_*’* is obtained. The thermal efficiency* η* of the torch corresponds to the ratio of *P*_*net*_ and *P*_*in*_.

There are basically two different approaches to achieving high plasma enthalpies, which can be combined:

- A thermally resilient design of the plasma torch,
- A suitable choice of plasma parameters.

They are briefly outlined in the following two subsections.

#### High-enthalpy torch concepts

High-enthalpy conditions are often associated with the (partial) use of nitrogen instead of argon as the primary plasma gas. However, nitrogen-based plasmas have not yet really caught on compared to argon-based plasmas. One reason for this may be that argon has the advantage of a low break-through potential, much lower than nitrogen or air, especially at atmospheric pressure^8^. This allows the arc discharge to be ignited at moderate voltages. This can be illustrated by means of the electrical conductivity. It is true that it is very similar for argon and nitrogen over a wide temperature range. However, if it is expressed as a function of the volumetric enthalpy *h’*, there are significant differences: to achieve a given electrical conductivity, significantly more energy is required for nitrogen than for argon. This is also true when the electrical conductivity is related to the mass specific enthalpy *h*^1^.

In addition, nitrogen-based plasmas are widely considered to be “electrode killers” because of the increased anode wear observed especially in older torches^9^, resulting in unstable and less efficient plasma characteristics^10^. The reason is that in a nitrogen-based plasma, the current density at the roots of the arc, and thus the local heat load, is significantly higher due to the greater constriction of the arc. Therefore, equipment manufacturers are trying to make their torch designs more suitable for nitrogen-based operation in order to move into the high enthalpy range. Here, a brief overview is given.

Legacy plasma guns such as the F4, 3MB, 7MB, 9MB (Oerlikon, formerly Sulzer Metco Inc., Westbury, NY, USA) and the SG-100 (Praxair TAFA, Concord, NH, USA) are based on the conventional high-current, low-voltage, gas-stabilized approach to gun design. Essentially, the majority of commercial plasma systems still belong to this group, although they have several drawbacks in terms of stability, deposition efficiency and electrode life (due to the high current).

In contrast, gas stabilized plasma guns based on the high-voltage, low-current approach provide a stable plasma and extended lifetime. Two examples designed with enhanced plasma enthalpies in mind are the Axial III™ plasma gun (Northwest Mettech Corp., Surrey, BC, Canada) and the 100HE® plasma torch (Progressive Surface, Grand Rapids, MI, USA).

Lima^9^ sprayed porous yttria-stabilized zirconia (YSZ) thermal barrier coatings (TBCs) by atmospheric plasma spraying (APS) using the Axial III™ gun at high powder feed rate (100 g/min) and deposition efficiency (70%). It is a gas-stabilized plasma torch with a set of three individual cathode–anode units (assembled together in a 120° geometry), thus providing three separate arcs that converge to form a unified plasma jet inside the torch. It is based on the patents granted to Ross and Burgess in 1991 and 1996^11,12^. The gun offers an axial injection of solid or liquid feedstocks and can be operated with argon-nitrogen–hydrogen plasmas up to a maximum of 150 kW of total electrical input power *P*_*in*_ with currents *I* between 90 and 250 A per electrode. Argon or nitrogen can be used as primary plasma gas.

The high-enthalpy 100HE® torch is a single anode/single cathode plasma gun designed to achieve high deposition rates and efficiencies. High enthalpy gas mixtures including N_2_ can be used with operating powers up to 105 kW. Feedstock powders can be processed in either axial, radial, or external injection modes. Curry et al. sprayed two YSZ powders at 280 g/min^13^. In addition to powders, suspension plasma spraying (SPS) and solution precursor plasma spraying (SPPS) feedstocks can be processed with feed rates in the range of 10–100 ml/minute. Suspension plasma sprayed thermal barrier coatings have been produced with the HE100 and different suspension parameters^14^.

In addition to gas stabilization, wall-stabilized cascaded plasma torches such as TriplexPro™ and SinplexPro™ (Oerlikon Metco Inc., Westbury, NY, USA) and Delta Plasma Torch (GTV GmbH, Luckenbach, Germany) are also operated in the low-current, high-voltage mode, but with lower plasma gas flow. They provide very stable argon-based plasmas, but allow only limited addition of molecular secondary plasma species.

The use of liquid carriers in SPS and SPPS increases the need for high-enthalpy plasma sources, since a significant amount of energy is required to heat and evaporate the liquid precursor, followed by heating and melting the suspended solid particles^15^. This was the motivation for the development of a water-stabilized plasma torch (WSP technology) developed at the Institute of Plasma Physics Institute of Plasma Physics of the Czech Academy of Sciences (IPP, Prague, Czech Republic), which provides a high-enthalpy plasma jets, making them particularly suitable for the processing of liquid feedstocks^16,17^. The latest hybrid WSP technology (WSP®-H 500, ProjectSoft HK a.s., Hradec Králové, Czech Republic) combines the principles of gas (GSP) and water (WSP) stabilization^18,19^ to produce a plasma jet of relatively low density *ρ* (about 2.7 g/m^3^), high mass-specific enthalpy *h* (272 MJ/kg), and high velocity (up to 7000 m/s)^17^. This is equivalent to a volumetric enthalpy *h’* of 0.73 MJ/m^3^. No argon is required for operation, nitrogen or hydrogen–nitrogen mixtures are possible. However, the partial stabilization with argon provides a benefit of increased plasma density^20^. The gun can be operated with up to 160 kW electrical input power *P*_*in*_.

In 2015, Belashchenko et al. developed a high-voltage, low-current torch concept that is based on combined wall and gas stabilization (C^+^ Plasma)^5,6^. The motivation was the stable and durable high-voltage generation of nitrogen-based plasmas with high enthalpy. The gun can be operated with up to 120 kW electrical input power *P*_*in*_ to achieve plasma enthalpies *h*_*0*_*’* of up to 60 kJ/sl. Above, a significant reduction in anode lifetime was observed.

This compilation shows that there are many approaches to constructing thermally resilient guns that are suitable for the use of nitrogen as the primary plasma gas, thus enabling high-enthalpy conditions. In this work, the Axial III™ plasma torch was used.

#### High-enthalpy plasma conditions

The work of Vardelle et al.^21^ is one example suggesting nitrogen as the primary plasma gas component. They compared measured plasma and particle characteristics using a single cathode torch (similar to F4) with a nitrogen-based plasma parameter on the one side and an argon-based parameter on the other side. The nozzle diameters were 6 mm and 8 mm, respectively. In each case, the hydrogen content was adjusted to achieve the same voltages at the same currents. The torch efficiency was found to be higher for the argon-based parameter. This work reported a first comprehensive set of measurements of the plasma velocity and temperature fields as well as the particle velocities and in-flight temperatures. The results showed higher plasma temperatures and slightly higher plasma velocities for the nitrogen-based parameter. Correspondingly, the particle velocities were measured to be higher in the nitrogen-based case. The measured particle surface temperatures however were similar, some slightly higher for the argon-based parameter. This might be due to the slightly higher hydrogen content in this composition suggesting a higher thermal conductivity of the plasma.

Marple et al.^22^ used a 9 MB plasma gun (Oerlikon, formerly Sulzer Metco Inc., Westbury, NY, USA) to investigate the processing and properties of YSZ TBCs using Nitrogen as plasma gas. Similar to Vardelle et al.^21^, he investigated a nitrogen/hydrogen and an argon/hydrogen plasma gas mixture and varied the plasma power. For the nitrogen-based case and the hottest spray conditions, increased deposition efficiencies and higher particle in-flight temperatures were found. However, the particle in-flight velocities were lower. This was suggested to be an effect of the lower total plasma gas flow and viscosity in this case. The results of further studies performed by Lima et al. with a 3MB torch^23^ and Guerreiro et al. with a 9MB torch^24^ also point in the direction of greater efficiency of the spraying process when nitrogen is used as the primary plasma gas.

Lima’s recent work^9^ on porous thermal barrier coatings (TBCs) using the Axial III™ gun was already mentioned earlier. By spraying a commercial YSZ feedstock at a powder feed rate of 100 g/min using an argon/nitrogen spray parameters at an electrical input power of *P*_*in*_ = 98 kW, a deposition efficiency (DE) value of 70% and a porosity of 14.2% ± 0.5% were achieved.

Curry et al.^25^ systematically studied the effect of process conditions on particle properties using an Axial III™ gun with different argon-nitrogen–hydrogen mixtures. The effect of increasing of the hydrogen content was ambivalent. At constant volumetric plasma gas flow, the measured particle temperatures and velocities were reduced although the net power increased. On the other hand, keeping the mass flow constant resulted in conversely higher particle temperatures but also in lower particle velocities; the net power increased significantly. In contrast, the effects of higher nitrogen levels were consistent in both cases. Particle temperatures increased while the velocities were reduced. The increase in net power was even higher than observed for the increased hydrogen contents. The comparison of different nitrogen contents at the same net power and two different plasma gas mass flow rates resulted in better thermal efficiencies for the high nitrogen parameters in all cases studied.

Belashchenko et al. reported on TBCs deposited by the C^+^ plasma torch concept^5,6^ using various nitrogen and argon-based plasma gas compositions, including an additional 20 to 30 volume percent hydrogen. In general, the C^+^ process is operated at high voltage levels of 240 to 270 V and relatively low currents below 400 to 450 A, resulting in volumetric enthalpies *h*_*0*_*’* above 30 kJ/sl and deposition rates of 50 to 150 g/min. It was found that for the same currents, the voltages were higher with the nitrogen-based plasmas resulting in higher electrical input powers *P*_*in*_ than with the argon-based plasmas. Consequently, the same trends were observed for the volumetric plasma enthalpies *h*_*0*_*’*. The thermal efficiencies *η* of nitrogen-based plasmas were better than those of argon-based compositions. In addition, increasing the hydrogen content resulted also in higher electrical input power *P*_*in*_ and improved the thermal efficiency *η* of the torch. As an example, segmented TBCs were sprayed using a nitrogen–hydrogen plasma with an electrical input power *P*_*in*_ of 102 kW resulting in a volumetric plasma enthalpy of 52 kJ/sl and a DE of 75% to 80%.

### Objectives

The objective of this work is to contribute to a better understanding of the effects on the plasma characteristics and the sprayed coatings when argon is replaced by nitrogen as the primary plasma gas component. Therefore, a ternary argon-based plasma parameter, a ternary nitrogen-based parameter, and a nitrogen-based parameter without any argon were studied. On the one hand, transport coefficients were calculated for a wide temperature range to validate the heating and acceleration capabilities of these compositions. In the experimental part, yttria coatings were produced by suspension plasma spraying with an Axial III™ gun. In addition, in-flight particle characteristics were measured. The computational and experimental results were correlated and used to critically evaluate the relevance of nitrogen in improving feedstock treatment and whether this is actually due to high plasma enthalpies.

## Methods and procedures

### Spray experiments

**Fig. 1:**
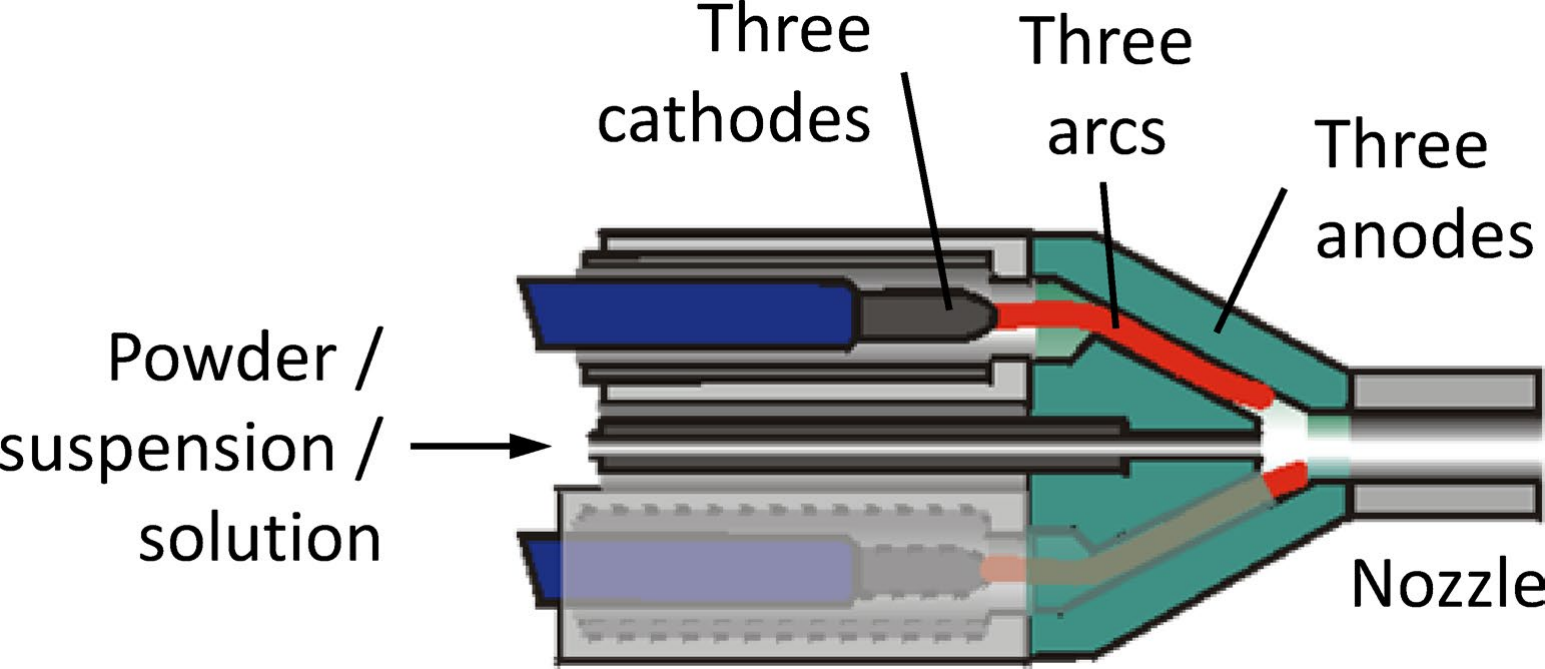
Schematic of the triple arc plasma generator Axial III™; adapted from^28^, licensed acc. to the terms of the Creative Commons Attribution Non-Commercial License CC BY-NC 3.0 (https://creativecommons.org/licenses/by-nc/3.0/).

Spray experiments were performed in the Jülich Thermal Spray Center (JTSC)^26^ using a commercial Axial III™ DC plasma torch^27^ (Northwest Mettech Corp., Surrey, BC, Canada) for suspension plasma spraying (SPS). This spray gun has a set of three single cathode–anode units spaced 120° apart, providing three separate arcs, see Fig. 1. It is based on a patent granted to Ross and Burgess in 1996^12^. The three independent DC arcs generate three plasma jets that converge inside the torch to form one unified plasma jet. Having three separate plasma generators enables central axial feedstock injection, which is advantageous for SPS because it can achieve high impact velocities on the substrate, thus limiting Stokes effects. The torch can operate at a total input power of up to 150 kW and use nitrogen instead of argon as the primary plasma gas. Two different nozzle diameters were used in this work, with diameters of 7.9 mm (5/16″) and 11.1 mm (7/16″).

The feedstock was an aqueous suspension AuerCoat® Y_2_O_3_ 30W T1 (Treibacher Industrie AG, Althofen, Austria) with 30% solid content (d_50_ ≈ 2.5 µm). The feed rate was 45 ml/min, one series of experiments was performed with 15 ml/min. The capillary for the central axial injection was 0.5 mm in diameter, and the atomizing gas flow was 6 sl/min argon. Average particle velocities and temperatures were measured with the Accuraspray 4.0 (Tecnar Automation Ltée, St. Bruno, QC, Canada) in a spray distance of 100 mm from the nozzle exit of the gun. The 4.0 unit was developed to achieve an improved better accuracy especially at SPS^29^. The manufacturer provides the following measurement accuracies: 3% for temperatures above 1000°C and 2% for velocities between 5 m/s and 1200 m/s^30^.

### Plasma parameter

A ternary argon-based plasma parameter, a ternary nitrogen-based parameter, and a nitrogen-based parameter without any argon were studied, details of which are given in Table 1. It should be noted that in all three cases the currents and the total plasma gas flows were the same.

**Table 1:** Plasma parameter for the spray experiments.

Case	I (A) per electrode	Ar (sl/min)	N₂ (sl/min)	H₂ (sl/min)	Total (sl/min)
Ar-based	220	165	33	22	220
		75%	15%	10%	
Ar-N	220	55	132	33	220
		25%	60%	15%	
N-based	220	0	165	55	220
		−	75%	25%	

Table 2 shows the net powers, thermal efficiencies and volumetric enthalpies obtained under these conditions. There are minor differences when comparing the data for the two different nozzles.

**Table 2:** Net powers, thermal efficiencies and volumetric enthalpies using two different nozzles.

Throat diameter	7.9 mm (5/16″)			11.1 mm (7/16″)		
Expansion ratio	3,24			1,65		
Case	**P**_**net**_** (kW)**	**therm. effic**	**h**_**0**_**’ (kJ/sl)**	**P**_**net**_** (kW)**	**therm. effic**	**h**_**0**_**’ (kJ/sl)**
Ar-based	35.7	36%	9.7	33.8	39%	9.2
Ar-N	63.9	48%	17.4	64.4	52%	17.6
N-based	72.8	50%	19.9	75.3	54%	20.5

### Calculations of thermodynamic and transport properties

The thermodynamic and transport properties being relevant to the operation of a plasma torch were obtained from Boulos et al.^4^. They were calculated at the Université de Limoges in France, and thoroughly validated against databases available at the University of Minnesota, USA, and the Université de Sherbrooke, Québec, Canada.

The densities were calculated by minimizing the Gibbs free energy in combination with the equations of mass conservation and electrical neutrality. The partition functions were determined by using the electronic energy levels and spectroscopic data from the literature and the more recent tables available from NIST. The calculated densities, partition functions and their derivatives were used to calculate the other thermodynamic properties such as specific enthalpy and heat capacity.

The transport properties were calculated using Sonine’s polynomial expansion of the first-order Chapman-Enskog approximation of the Boltzmann equation for the viscosity, the second-order approximation for thermal conductivity (excluding the electron contribution), and the third-order approximation for the electron thermal conductivity and electrical conductivity.

These tabulated data for argon, hydrogen, and nitrogen being relevant to this work and some others cover temperatures between 400 and 24,000 K in increments of 100 K. In addition, data are also available for argon–hydrogen mixtures. The pressure is always 100 kPa.

The thermodynamic and transport properties for the three plasma gas mixtures studied in this work (Table 1) have been calculated from the data for the individual components: argon, nitrogen, and hydrogen. Their proportions can be defined in terms of masses *m*_*i*_ or moles *n*_*i*_. When the property is related to mass, such as for the thermodynamic properties, i. e. density, specific heat, and enthalpy, the mass proportions *w*_*i*_ are appropriate:

1 $$w_{i} = \frac{{m_{i} }}{{\mathop \sum \nolimits_{i} m_{i} }}$$

The transport properties of mixtures however, depend on the molar fractions *x*_*i*_:

2 $$x_{i} = \frac{{n_{i} }}{{\mathop \sum \nolimits_{i} n_{i} }}$$

If molecular gases are components of the plasma in combination with atomic gases, this equation is applicable as long as there is no dissociation of the molecular species. At higher temperatures above the onset of dissociation, the number of moles is multiplied, e.g. doubled in the case of a diatomic gas. Since dissociation already occurs at relatively low temperatures in plasma technologies, Gleizes et al.^31^ proposed the ‘dissociated mole fraction’ *y*_*i*_:

3 $$y_{i} = \frac{{j_{i} n_{i} }}{{\mathop \sum \nolimits_{i} j_{i} n_{i} }}$$

where *j*_*i*_ is one for atomic gases and the number of atoms in a gas molecule before dissociation if they are molecular. This is also used in this paper for nitrogen and hydrogen.

The thermodynamic properties of plasma gas mixtures have been calculated as follows:

4 $$Density:\quad \rho = \left( {\mathop \sum \limits_{i} \frac{{w_{i} }}{{\rho_{i} }}} \right)^{ - 1}$$

5 $$Mass - specific \, enthalpy:\quad h = \mathop \sum \limits_{i} w_{i} h_{i}$$

Different mixing rules were tried for the transport properties. First, the dynamic viscosities, thermal and electrical conductivities were calculated for a mixture containing equal molar proportions of argon and hydrogen. The results were then compared with the tabulated data of Boulos et al.^4^ mentioned before.

For the dynamic viscosity *η*, the well-known approach of Wilke^32^, based on the physical significance of the elastic collision in a plasma, gave a very good approximation to the reference data:

6 $$\eta = \mathop \sum \limits_{i} \frac{{x_{i} \eta_{i} }}{{1 + \mathop \sum \nolimits_{k \ne i} Z_{ik} x_{k} }}$$

7 $$Z_{{ik}} = \frac{1}{{2\sqrt 2 }}\left( {1 + \frac{{M_{i} }}{{M_{k} }}} \right)^{{ - {\raise0.7ex\hbox{$1$} \!\mathord{\left/ {\vphantom {1 2}}\right.\kern-\nulldelimiterspace} \!\lower0.7ex\hbox{$2$}}}} \left[ {1 + \left( {\frac{{\eta _{i} }}{{\eta _{k} }}} \right)^{{{\raise0.7ex\hbox{$1$} \!\mathord{\left/ {\vphantom {1 2}}\right.\kern-\nulldelimiterspace} \!\lower0.7ex\hbox{$2$}}}} \left( {\frac{{M_{k} }}{{M_{i} }}} \right)^{{{\raise0.7ex\hbox{$1$} \!\mathord{\left/ {\vphantom {1 4}}\right.\kern-\nulldelimiterspace} \!\lower0.7ex\hbox{$4$}}}} } \right]^{2}$$

where *M*_*i*_ are the molecular masses and *η*_*i*_ are the dynamic viscosities of the gas components *i*. The factors *Z*_*ik*_ are the Sutherland coefficients which are dimensionless and independent of the composition of the mixture. They take into account the different molecular weights of the components in the gas mixture, since the viscosity of gases depends highly on these.

Wilke’s approach is not suitable for calculating thermal conductivities *λ* of mixtures because it is based on the physics of elastic collisions. For the thermal conductivity, however, inelastic collisions (the reactional part of the thermal conductivity) contribute a large portion of the total thermal conductivity^31^. The approaches proposed by Lindsey and Bromley^33^ and Mathur et al.^34^ have been tried, as well as a simple linear interpolation in terms of the ‘dissociated molar fractions’ *y*_*i*_. However, the best agreement was obtained with the equation of Mason and Saxena^35,36^. It is based on the Wassiljewa formula^37^ and has also been recommended by other authors^4,38^. In this work it is used with the ‘dissociated molar fractions’ *y*_*i*_:

8 $$\lambda = \mathop \sum \limits_{i} \frac{{\lambda_{i} }}{{1 + \mathop \sum \nolimits_{k \ne i} G_{ik} \frac{{y_{k} }}{{y_{i} }}}}$$

9 $$G_{{ik}} = \frac{{1.065}}{{2\sqrt 2 }}\left( {1 + \frac{{M_{i} }}{{M_{k} }}} \right)^{{ - {\raise0.7ex\hbox{$1$} \!\mathord{\left/ {\vphantom {1 2}}\right.\kern-\nulldelimiterspace} \!\lower0.7ex\hbox{$2$}}}} \left[ {1 + \left( {\frac{{\eta _{i} M_{k} }}{{\eta _{k} M_{i} }}} \right)^{{{\raise0.7ex\hbox{$1$} \!\mathord{\left/ {\vphantom {1 2}}\right.\kern-\nulldelimiterspace} \!\lower0.7ex\hbox{$2$}}}} \left( {\frac{{M_{i} }}{{M_{k} }}} \right)^{{{\raise0.7ex\hbox{$1$} \!\mathord{\left/ {\vphantom {1 4}}\right.\kern-\nulldelimiterspace} \!\lower0.7ex\hbox{$4$}}}} } \right]^{2}$$

The factor 1.065 is an indication of the semi-empirical nature of this approach. The agreement with the tabulated reference data of the argon–hydrogen mixture was good.

When the ionization energies of the gas components involved in are similar (as for argon, nitrogen and hydrogen), a simple linear interpolation using molar fractions is sufficient to calculate the electrical conductivity *σ* of a mixture^31^. In this work, the ‘dissociated molar fractions’ *y*_*i*_ have been used:

10 $$\sigma = \mathop \sum \limits_{i} y_{i} \sigma_{i}$$

The agreement with the tabulated reference data of the argon–hydrogen mixture was very good.

## Results and discussion

### Properties of elemental plasma gases

In order to elucidate the influence of the individual gas species on the properties of the gas mixtures, the thermodynamic and transport properties of argon, nitrogen and hydrogen were calculated first.

**Fig. 2:**
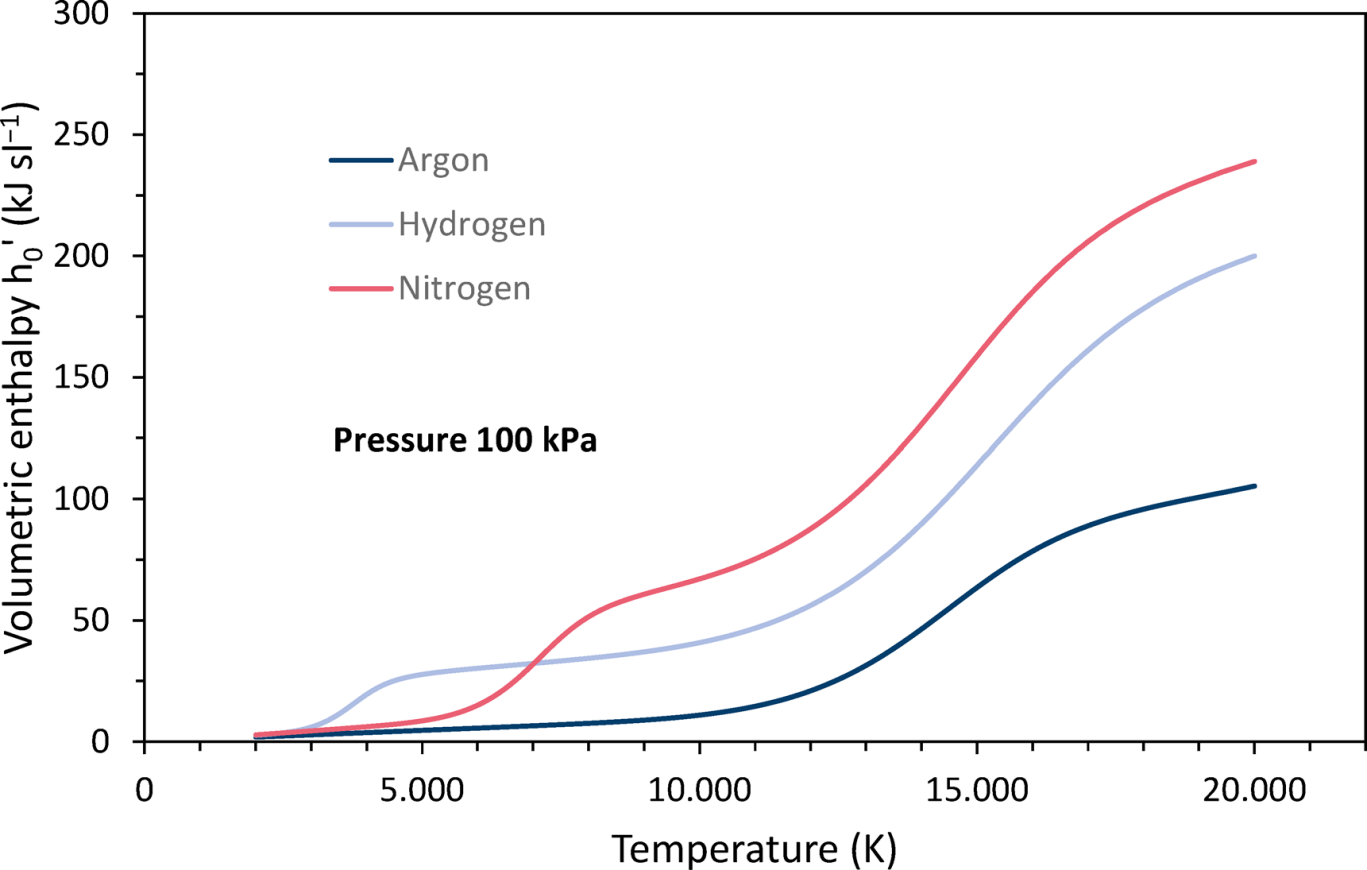
Temperature dependence of the volumetric enthalpies h_0_’ of argon, nitrogen and hydrogen at 100 kPa pressure, calculated based on data of Boulos et al.^4^.

The volumetric enthalpy *h*_*0*_*’* was calculated according to

11 $$h_{0} ^{\prime } = h\rho _{0}$$

where *h* is the mass-specific enthalpy given as function of the temperature in^4^ and *ρ*_*0*_ is the mass density at STP conditions. Figure 2 shows the temperature dependence for argon, nitrogen and hydrogen. The shape of the curves is due to the different characteristics of the mass-specific enthalpies and mass densities. On the one hand, the mass-specific enthalpies increase linearly with temperature, superimposed by increases due to the uptake of the dissociation energy for hydrogen around 3700 K and nitrogen around 7000 K, and of the ionization energy for argon around 14,800 K, nitrogen around 14,500 K, and hydrogen around 14,700 K. On the other hand, the densities decrease degressively, superimposed by slight dips due to the dissociation of hydrogen and nitrogen. Thus, the resulting plots show peaks due to both dissociation and ionization. Above 6300 K, nitrogen has the largest volumetric enthalpy.

**Fig. 3:**
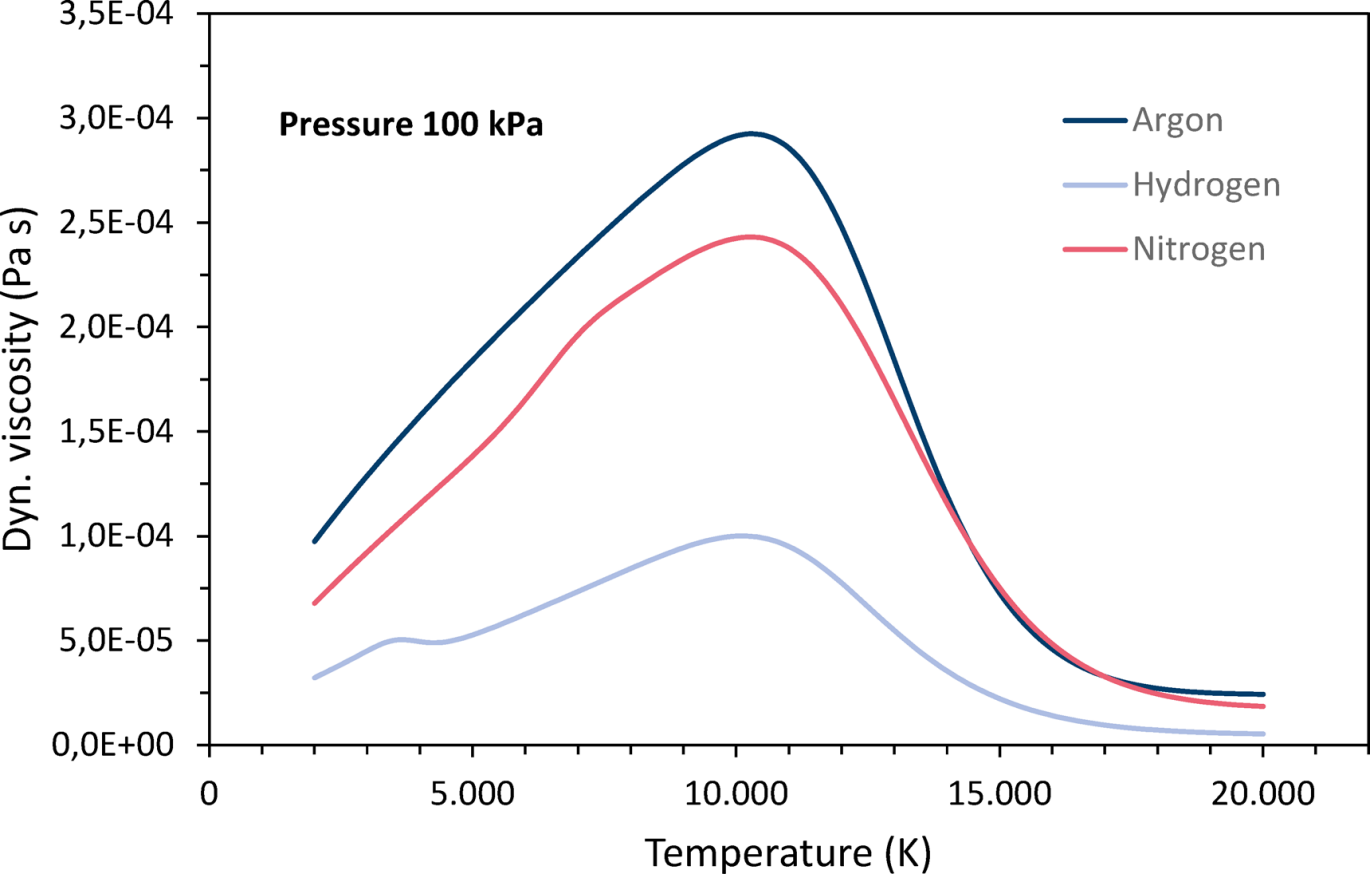
Temperature dependencies of the dynamic viscosities of argon, nitrogen and hydrogen at 100 kPa pressure, data taken from Boulos et al.^4^.

The temperature dependence of the dynamic viscosities *η* is shown in Fig. 3. Initially, the viscosities increase continuously with increasing temperature. For hydrogen and nitrogen, very small peaks are superimposed on the overall increase as they dissociate. The viscosity increases up to the temperatures where the collision of neutrals reaches its maximum^39^ and ionization begins, for hydrogen at about 10,100 K, for argon and nitrogen at about 10,300 K. With increasing ionization, long-range Coulomb interactions between charged particles reduce the interactions between the gas particles and thus, the viscosity decreases^1^. The downward trend ends when ionization is complete. Argon and nitrogen have the highest viscosities, while hydrogen has much lower values.

**Fig. 4:**
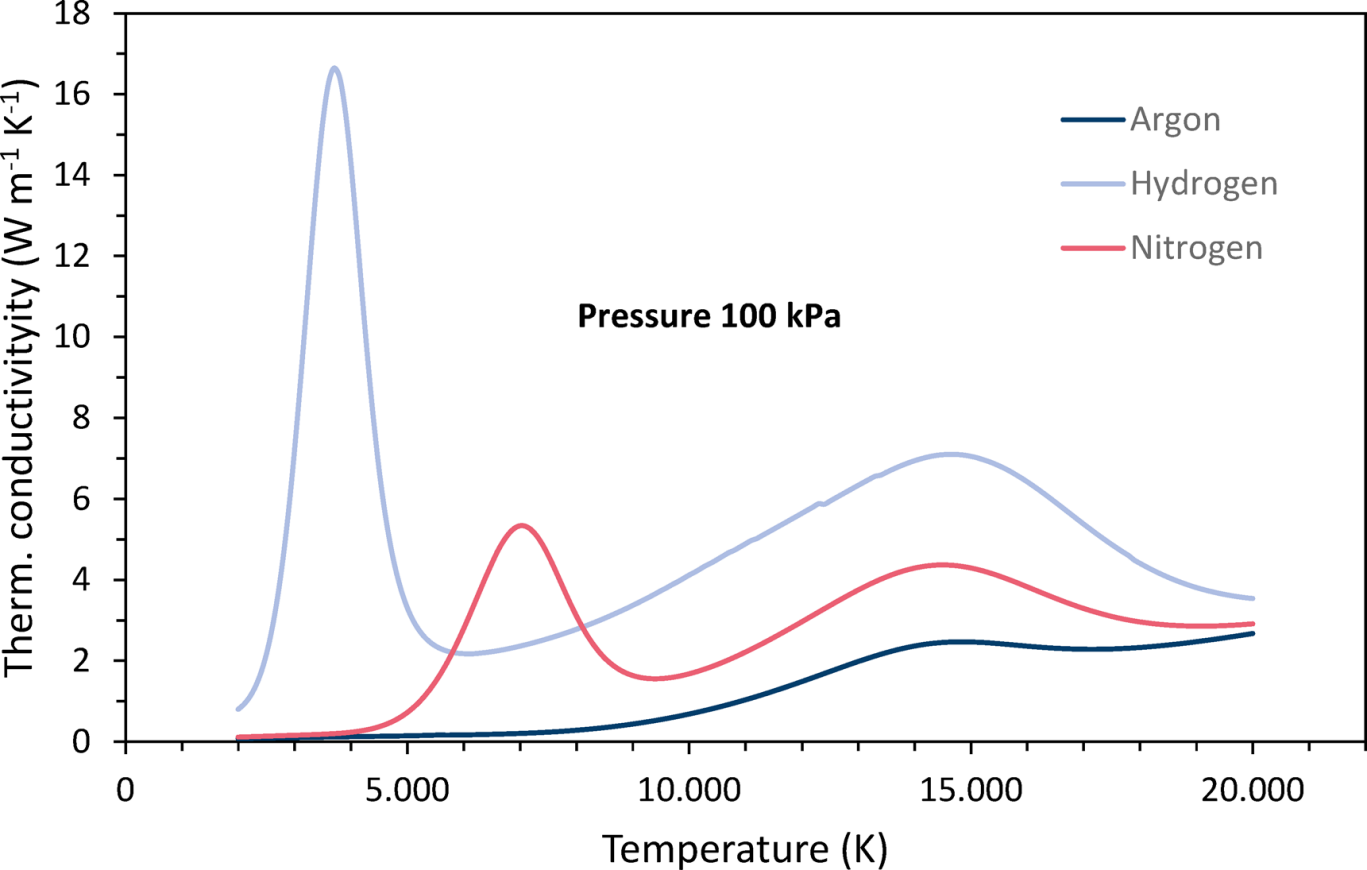
Temperature dependencies of the thermal conductivities of argon, nitrogen and hydrogen at 100 kPa pressure, data taken from Boulos et al.^4^.

The temperature dependence of the thermal conductivities *λ* is shown in Fig. 4. The pronounced peaks in the temperature curves are associated with both dissociation and ionization. It is the reactional part of the thermal conductivity that is based on these reactions. Note the high peak for hydrogen dissociation at relatively low temperatures, followed by the peak for nitrogen dissociation. Hydrogen has the highest overall thermal conductivity in almost the entire temperature range.

**Fig. 5:**
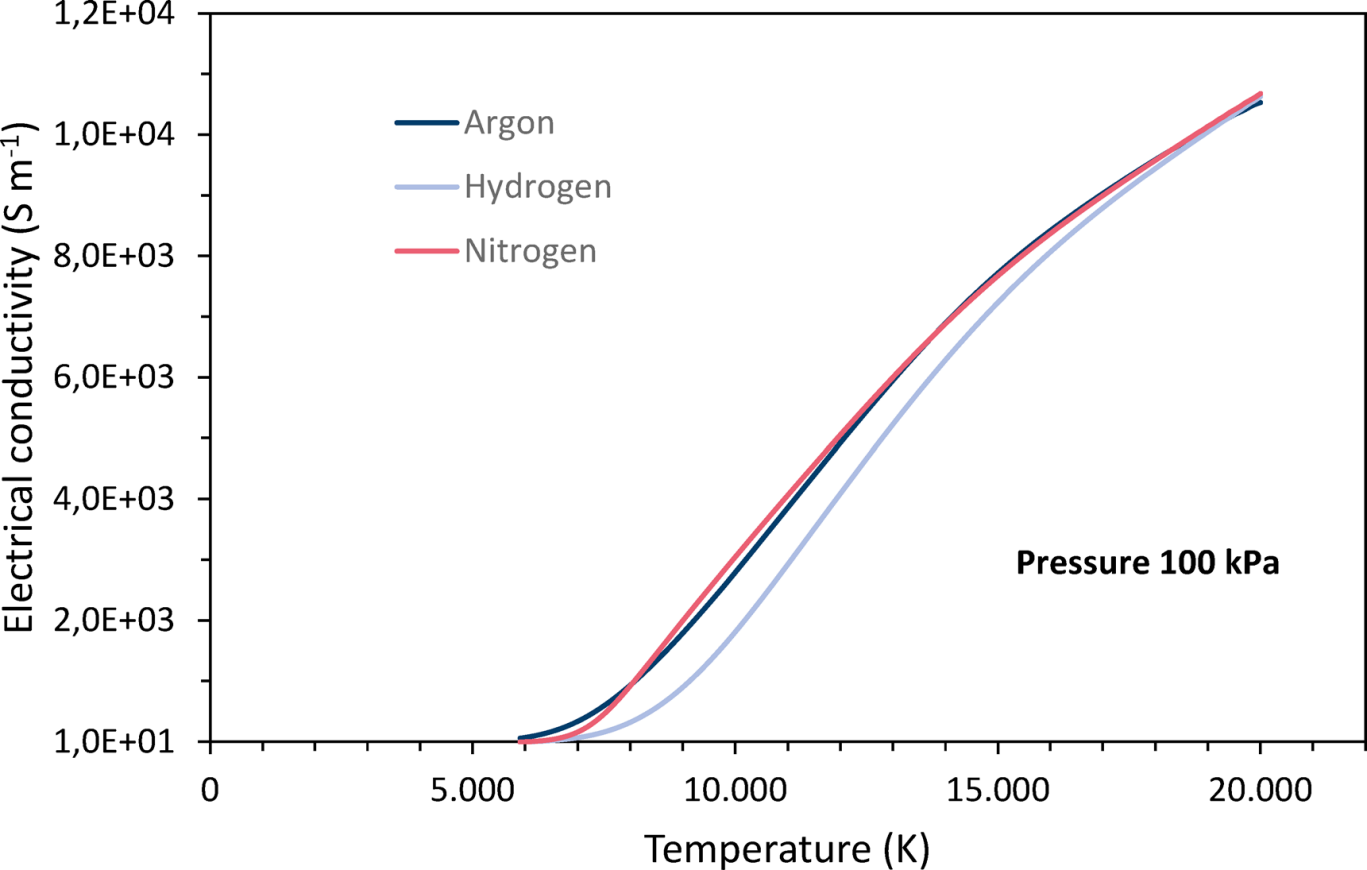
Temperature dependencies of the electrical conductivities of argon, nitrogen and hydrogen at 100 kPa pressure, data taken from Boulos et al.^4^.

The electrical conductivity *σ* depends mainly on the electron density which varies almost exponentially with the temperature^4^. Since argon, nitrogen and hydrogen have quite similar ionization potentials, their electrical conductivities are comparable, for argon and nitrogen almost identical and slightly lower for hydrogen, see Fig. 5. They increase gradually with increasing temperature until the ionization is most intense. At even higher temperatures, the slope decreases again. Because the electrical conductivities are so similar, they are not considered further in this paper.

### Properties of plasma gas mixtures

The thermodynamic and transport properties of the three plasma gas mixtures used in this work listed in Table 1 were calculated applying the mixing rules described earlier in section “Calculations of Thermodynamic and Transport Properties”.

**Fig. 6:**
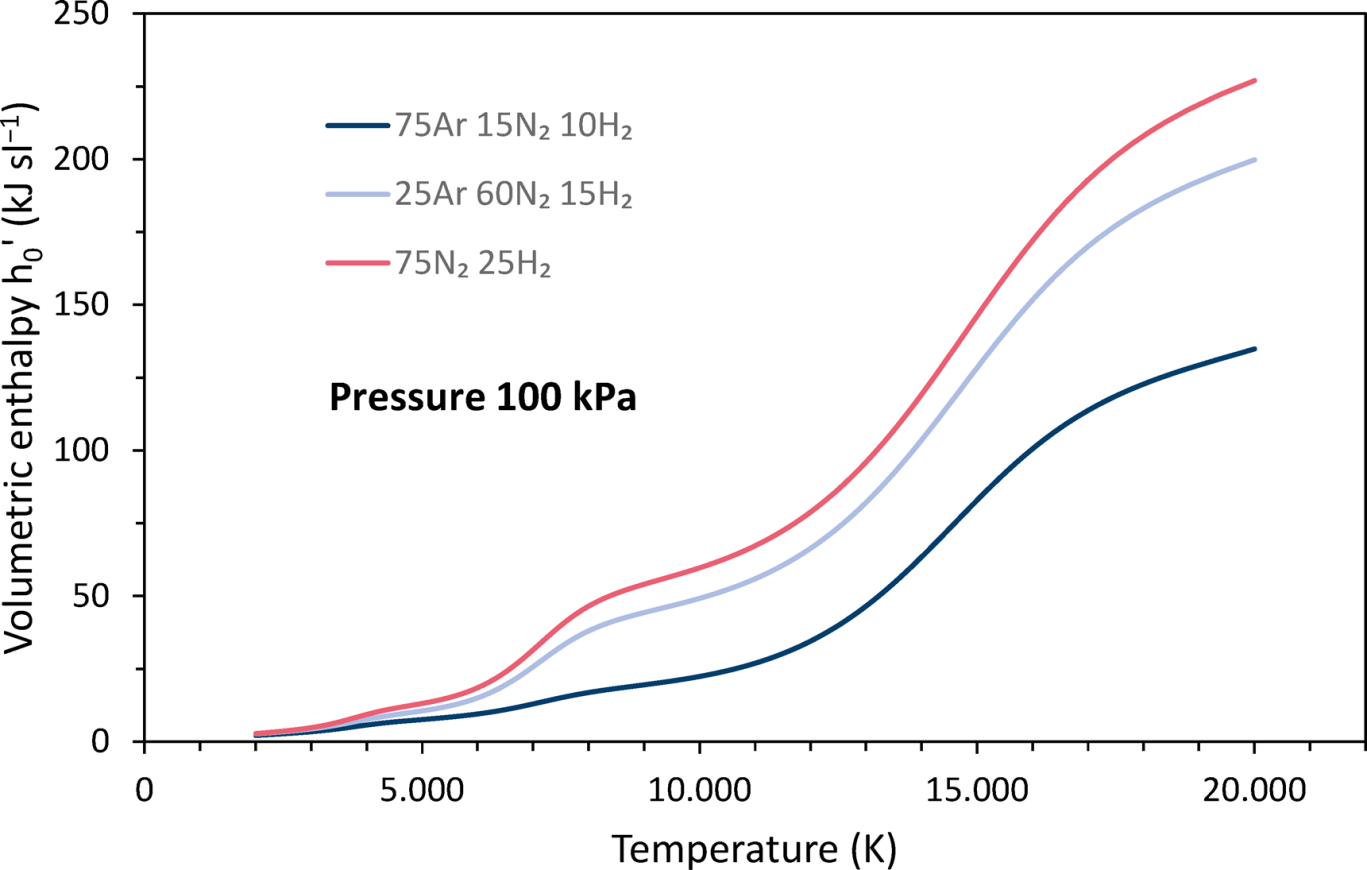
Volumetric enthalpy enthalpies h_0_’ for the three gas mixtures as functions of the temperature.

Figure 6 shows the volumetric enthalpies *h*_*0*_*’* for the three gas mixtures as a function of temperature. As mentioned before, they were calculated under the assumption that the entire net power of the torch was transferred to the plasma. The increases due to dissociation and ionization vary in magnitude depending on the proportions of the corresponding gases. The highest volumetric enthalpy is for the N-based parameter.

**Fig. 7:**
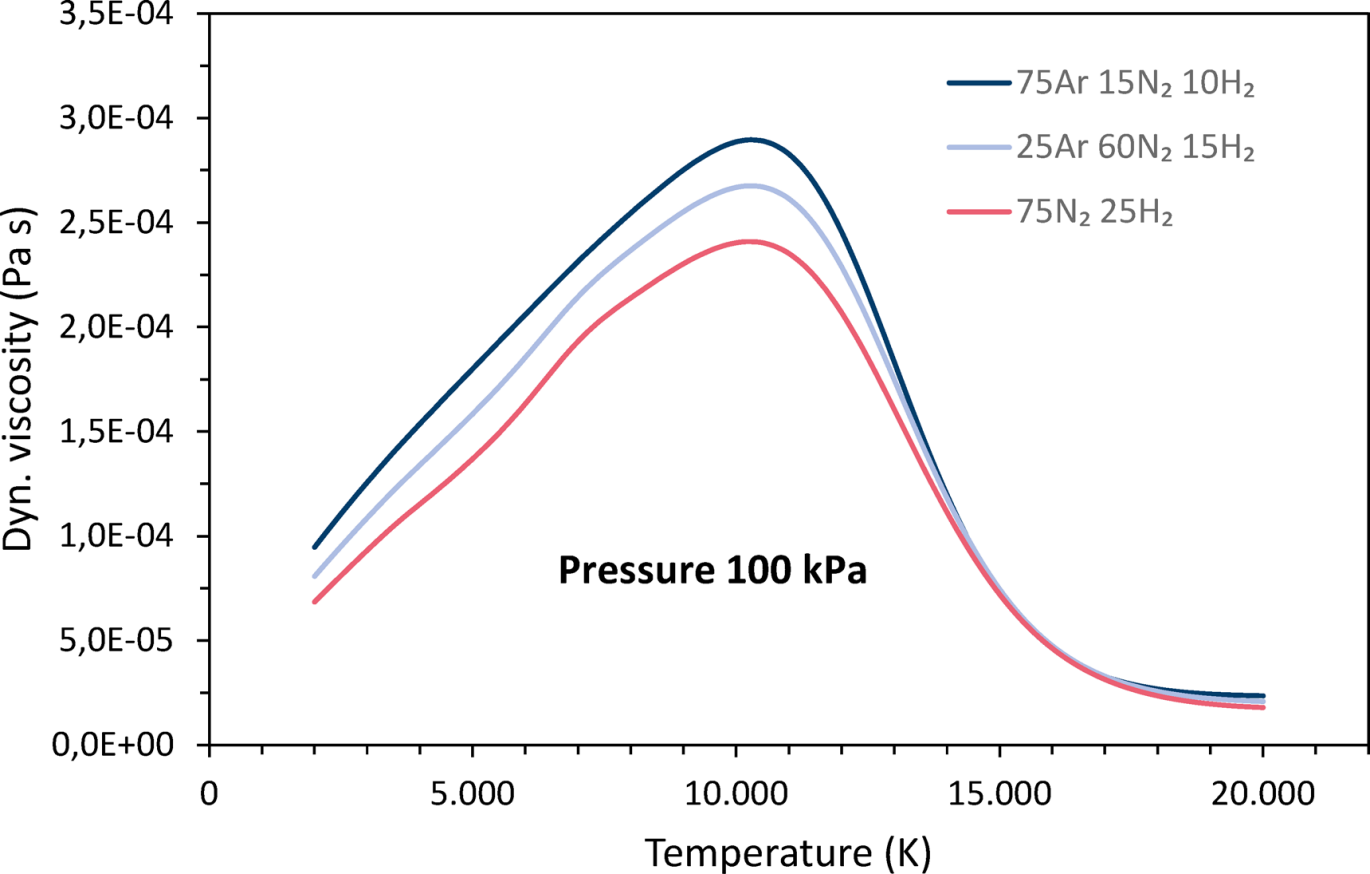
Dynamic viscosities for the three gas mixtures as functions of the temperature.

Figure 7 shows the dynamic viscosities for the three gas mixtures as a function of temperature. The viscosities are higher with increasing argon content and decreasing nitrogen content. The overall differences are relatively small.

**Fig. 8:**
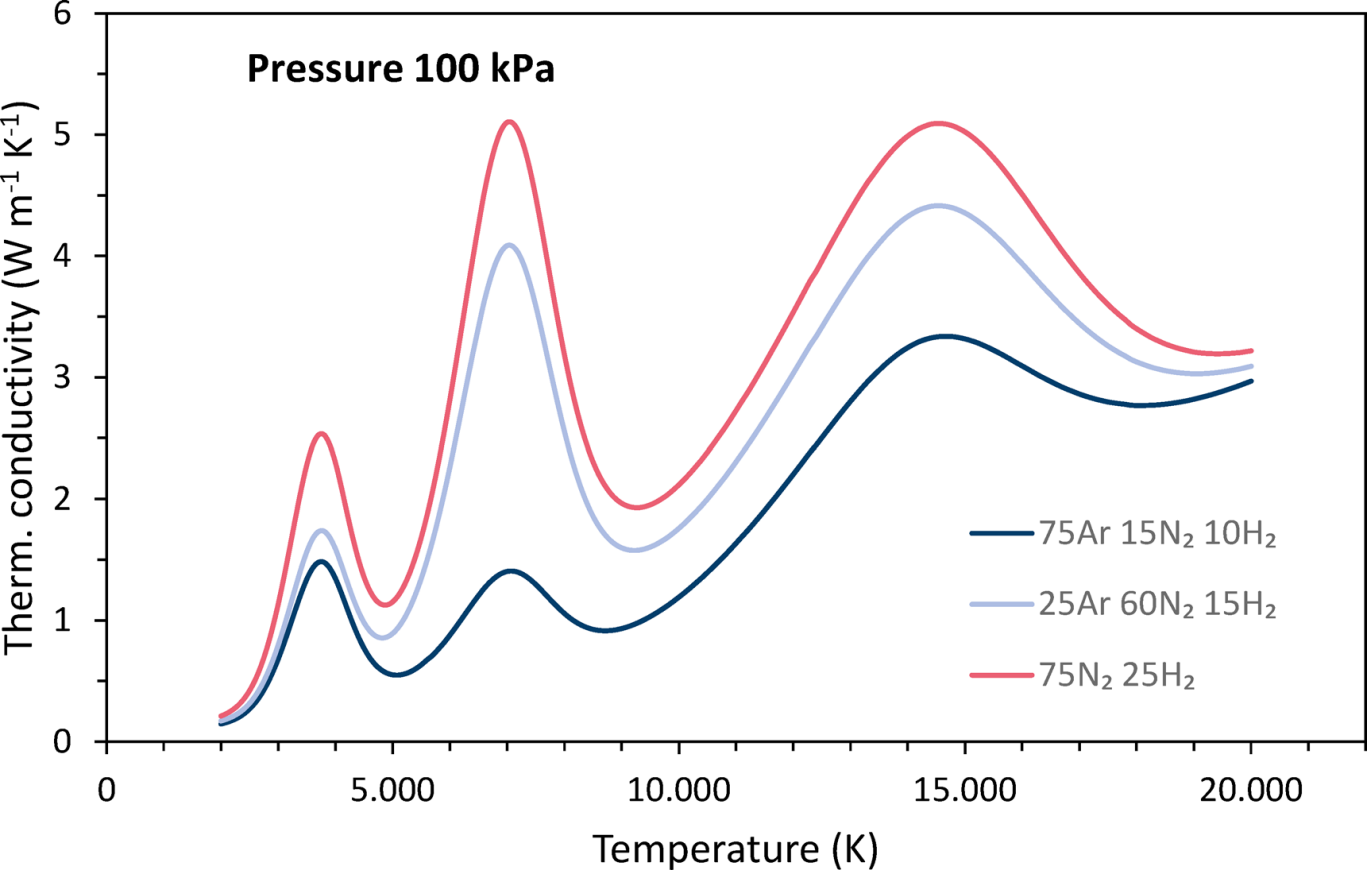
Thermal conductivities for the three gas mixtures as functions of the temperature.

Figure 8 shows thermal conductivities for the three gas mixtures as a function of temperature. The peaks due to dissociation and ionization vary in magnitude depending on the proportions of the corresponding gases. The hydrogen content has the greatest effect on the increase.

### Plasma properties at spray conditions

**Fig. 9:**
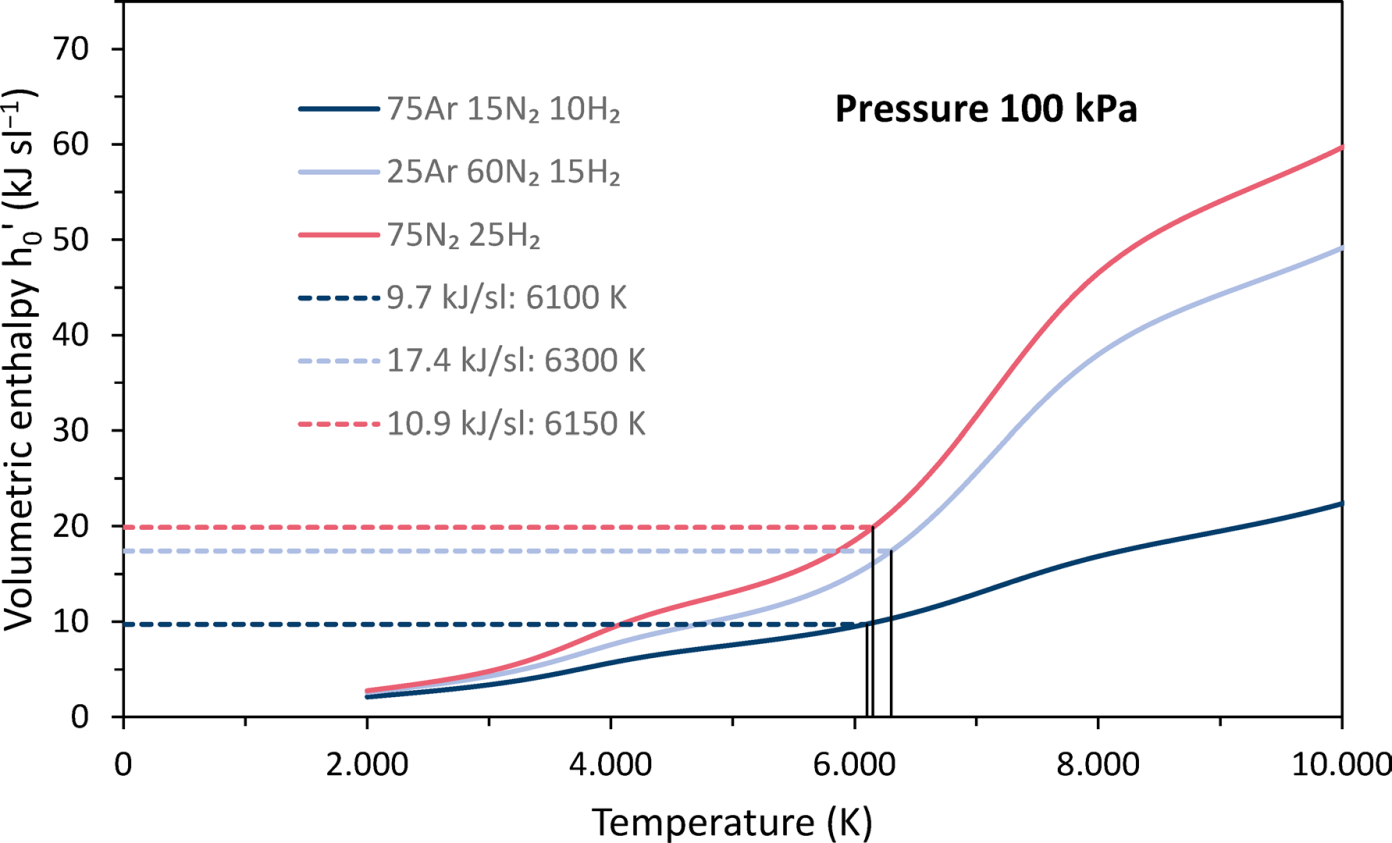
Volumetric enthalpies h_0_’ for the three gas mixtures as functions of the temperature (section of Fig. 6); volumetric enthalpy levels (horizontal lines) and identified plasma temperatures (vertical lines) are plotted for the 7.9 mm (5/16″) nozzle.

Figure 9 shows a section of Fig. 6 with the volumetric enthalpies *h*_*0*_*’* for the three gas mixtures as functions of the temperature. In addition, the volumetric enthalpy levels are plotted, which were obtained for the three different plasma parameters with the 7.9 mm (5/16″) nozzle, see Table 2.

Slightly different levels were obtained for the 11.1 mm (7/16″) nozzle (see Table 2, not plotted in Fig. 9). These allow the corresponding plasma temperatures to be identified. They are assumed to be the average temperatures in the entire flow channel of the plasma when the net power is fully transferred to the plasma before exiting the nozzle. The identified temperatures are listed in Table 3. In addition, some thermodynamic and transport properties at these temperatures have been calculated for the three mixtures and are also included. There are minor differences when comparing the data for the two different nozzles.

**Table 3:** Identified plasma temperatures, mass densities, dynamic viscosities and thermal conductivities using two different nozzles.

	7.9 mm (5/16″)				11.1 mm (7/16″)			
Case	T (K)	Density (kg m^−3^)	Dyn. vis. (Pa s)	Th. cond. (W m^−1^ K^−1^)	T (K)	Density (kg m^−3^)	Dyn. vis. (Pa s)	Th. cond. (W m^−1^ K^−1^)
Ar-based	6100	6.04∙10^−2^	2.09∙10^−4^	0.94	5900	6.28∙10^−2^	2.03∙10^−4^	0.82
Ar-N	6300	4.08∙10^−2^	1.94∙10^−4^	2.96	6200	4.20∙10^−2^	1.91∙10^−4^	2.72
N-based	6150	3.17∙10^−2^	1.68∙10^−4^	3.24	6200	3.13∙10^−2^	1.69∙10^−4^	3.39

Under the boundary conditions of this study, i.e. equal currents and equal total gas flows for all three parameters, it can be seen that although an increasing nitrogen content causes higher plasma enthalpies, the plasma temperatures are very similar to those at a low nitrogen content. Obviously, there is no advantage of a high nitrogen content in terms of the plasma temperature achieved. This is a result of the interaction of the specific enthalpy of the plasma gas composition with the increasing voltage and thermal efficiency that occur in constant current mode.

The enthalpy describes the amount of energy that is stored in the plasma gas for a given unit of volume or mass that can be released again. A high specific plasma enthalpy is not in itself an advantage. In plasma spraying, the plasma gas’s enthalpy is only a limiting factor as long as it is not much more than the energy needed to heat the feedstock to the desired temperature. In this case, load effects are observed, e.g. at very high feed rates of solid or liquid feedstocks.

Belashchenko et al.^5^ see another advantage of nitrogen-based plasmas in the fact that undesirably high power losses due to plasma radiation are only reached at significantly higher specific enthalpies than in argon-based plasmas. However, it has already been stated that high specific enthalpies are not in themselves an advantage. Belashchenko estimated a temperature threshold of about 12,000 K, above which the operation of a typical plasma torch becomes inefficient due to excessive radiation losses in the order of 10 kW. However, when comparing the property data compiled by Boulos^4^ for this temperature, argon has a total volumetric emission coefficient of about 10^9^ W/m^3^ and nitrogen one order of magnitude higher. Thus, for a given temperature, argon appears to be more advantageous in terms of lower radiation losses. Hydrogen and argon have similar emission coefficients.

### Particle acceleration

The data in Table 3 can be used to calculate characteristic quantities that determine the particle acceleration ability of the plasma.

The drag force that is exerted by the plasma gas flow on a spherical particle is given by

12 $$F_{D} = \frac{\pi }{8}d_{P}^{2} \rho v_{rel}^{2} C_{D}$$

where *d*_*p*_ is the particle diameter, *ρ* is the gas density, *v*_*rel*_ is the relative velocity between fluid and particle, and *C*_*D*_ is the drag coefficient. For small Reynold numbers, i.e. for the Stokes’ regime, the latter can be set to

13 $$C_{D} = \frac{24}{{Re}}\quad {\text{with}}\;{\text{the}}\;{\text{Reynold}}\;{\text{number}}\quad Re = \frac{{d_{P} \rho \left| {v_{rel} } \right|}}{\eta }$$

where *η* is the dynamic viscosity of the fluid. Assuming that the particle velocity is very small near the injection point, the relative velocity can be set equal to the absolute fluid velocity *v*, which is given by

14 $$v = \frac{{Q_{0} }}{{A_{torch} }} \frac{{\rho_{0} }}{\rho }$$

where *Q*_0_ is the total gas supply to the torch at STP, *A*_*torch*_ is the torch channel cross-sectional area, and *ρ*_0_ is the gas density at STP conditions.

Combining these three equations and assuming a constant particle diameter *d*_*p*_, gas supply *Q*_0_, and torch channel cross-section *A*_*torch*_, the drag force has the proportionality

15 $$F_{D} \propto \eta \frac{{\rho_{0} }}{\rho } \equiv AAF$$

which is defined in this work as the Acceleration Ability Factor (AAF). It is expressed in units of the dynamic viscosity.

**Fig. 10:**
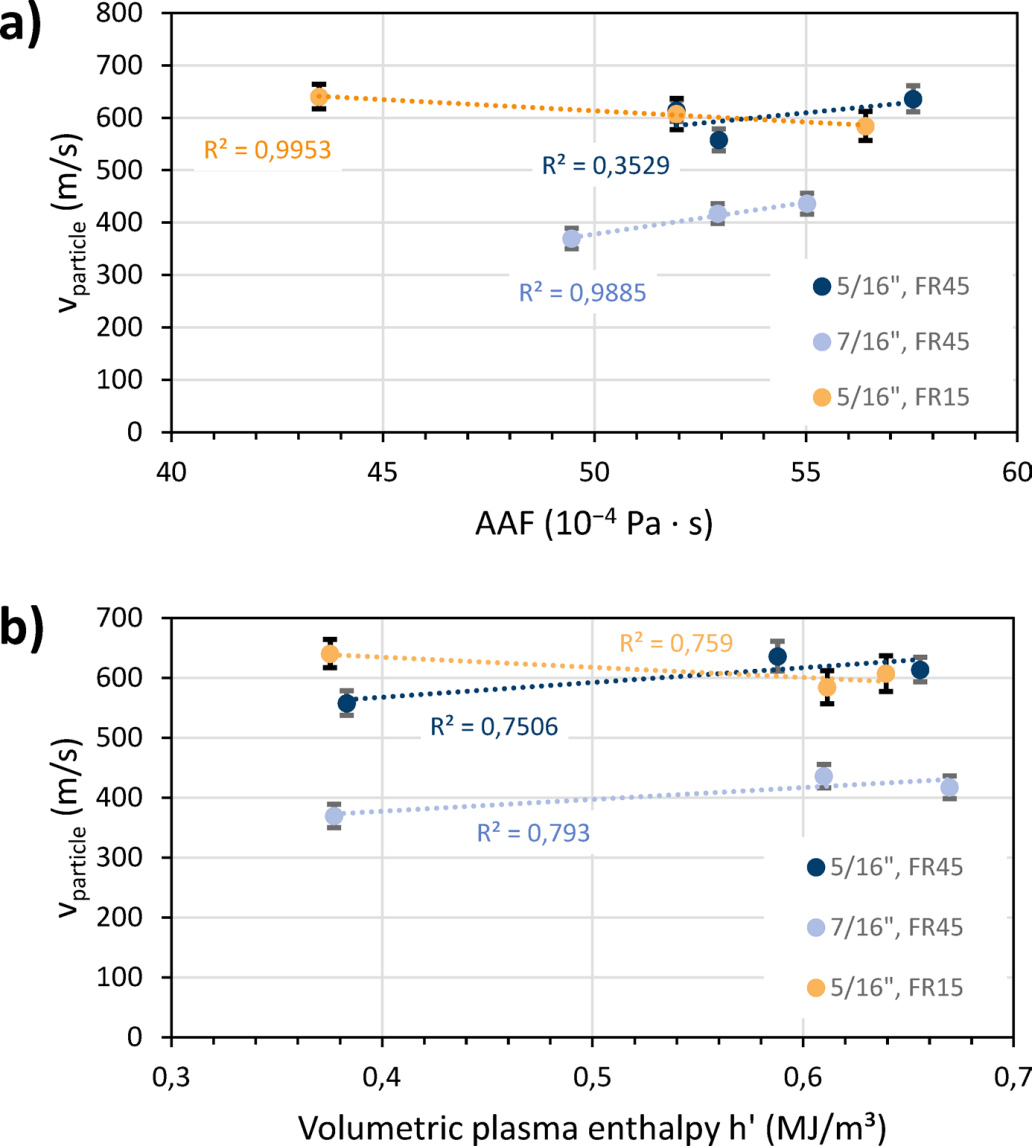
Average measured in-flight velocities of the particles for the three plasma gas compositions, each for the 7.9 mm (5/16″) and the 11.1 mm (7/16″) nozzles, (**a**) plotted as function of the AAF, (**b**) plotted as function of the volumetric enthalpy at 100 kPa; the FR labels indicate the suspension feed rates of 15 ml/min and 45 ml/min, and the error bars show the temporal variation of the measured velocities.

Figure 10a shows the average measured in-flight velocities of the particles for the three plasma gas compositions, each for the 7.9 mm (5/16″) and the 11.1 mm (7/16″) nozzles. The average temporal variation of the measured velocities was found to be ± 4.3%, which is greater than the 2% measurement accuracy. Two suspension feed rates were set for the smaller nozzle: 15 ml/min and 45 ml/min. The velocities were plotted as a function of the AAF, which was calculated from the Table 3 data. The viscosity *η* and the density *ρ* describe the conditions in the immediate vicinity of the nozzle exit, where the interaction between the plasma gas and the particles predominantly occurs. As these variables constitute the AAF in conjunction with *ρ*_0_ , they act in opposite directions. An increase in nitrogen content results in a slight increase in the density ratio *ρ*_0_/*ρ* and a slight decrease in dynamic viscosity *η*. Therefore, the AAF changes only insignificantly for the examined cases, and the measured speeds behave similarly. The highest particle velocities were obtained at the highest AAFs for both nozzles with the Ar-N parameter and the lowest velocities were obtained with the N-based parameter (7.9 mm [5/16″] nozzle) and the Ar-based parameter (11.1 mm [7/16″] nozzle), respectively (see Table 1). Naturally, overall higher velocities were achieved with the smaller nozzle diameter and higher expansion ratio.

Figure 10b shows the same particle velocity data, but here plotted as a function of the volumetric plasma enthalpy *h’*, reflecting the conditions at the actual plasma temperature. Although the enthalpy varied somewhat more than the AAF for the cases examined, the velocities behaved virtually indifferently.

### Particle heating

Particle heating and melting can be estimated based on the considerations of Engelke^40,41^. He equated the time required for a particle to reach the substrate at the spray distance *s* through the plasma jet with the time required for this particle to melt completely^42^. Assuming a constant plasma temperature during the particle flight, a purely conductive energy transfer mechanism (Nusselt number Nu = 2), and by introducing the simplification with respect to the Biot number $2/\left(1+2/Bi\right)\approx Bi$, the separation of the plasma and particle properties yields the expression

16 $$\left( {T - T_{RT} } \right)^{2} \frac{{\lambda^{2} s}}{{\eta v_{rel} }} = \frac{{h_{p}^{2} d_{p}^{2} \rho_{p} }}{16}$$

In this equation, the plasma-related parameters are on the left and the particle-related parameters are on the right. *λ* is thermal conductivity of the plasma, *T* is the temperature of the plasma, *T*_*RT*_ is the room temperature, *s* is the spray distance, *η* is the dynamic viscosity of the plasma, *v*_*rel*_ is the relative velocity between the plasma and the particle, *d*_*p*_ is the particle diameter, and *ρ*_*p*_ is the mass density of the particle. *h*_*p*_ is the total enthalpy of the particle including the latent heat of fusion *h*_*m*_

17 $$h_{p} = \mathop \smallint \limits_{{T_{RT} }}^{T} c_{p} \left( T \right)dT + h_{m}$$

where *c*_*p*_ is the specific heat of the particle.

Assuming constant temperatures, velocities and spray distances, the left side of this equation has the following proportionality which was defined as the Ability of Heating Factor AHF^40,41^

18 $$\frac{{\lambda^{2} }}{\eta } \equiv AHF$$

In this work, a modified form of the AHF is used, as proposed by Pateyron^43^

19 $$AHF^{\prime} = \frac{\lambda }{\sqrt \eta }$$

**Fig. 11:**
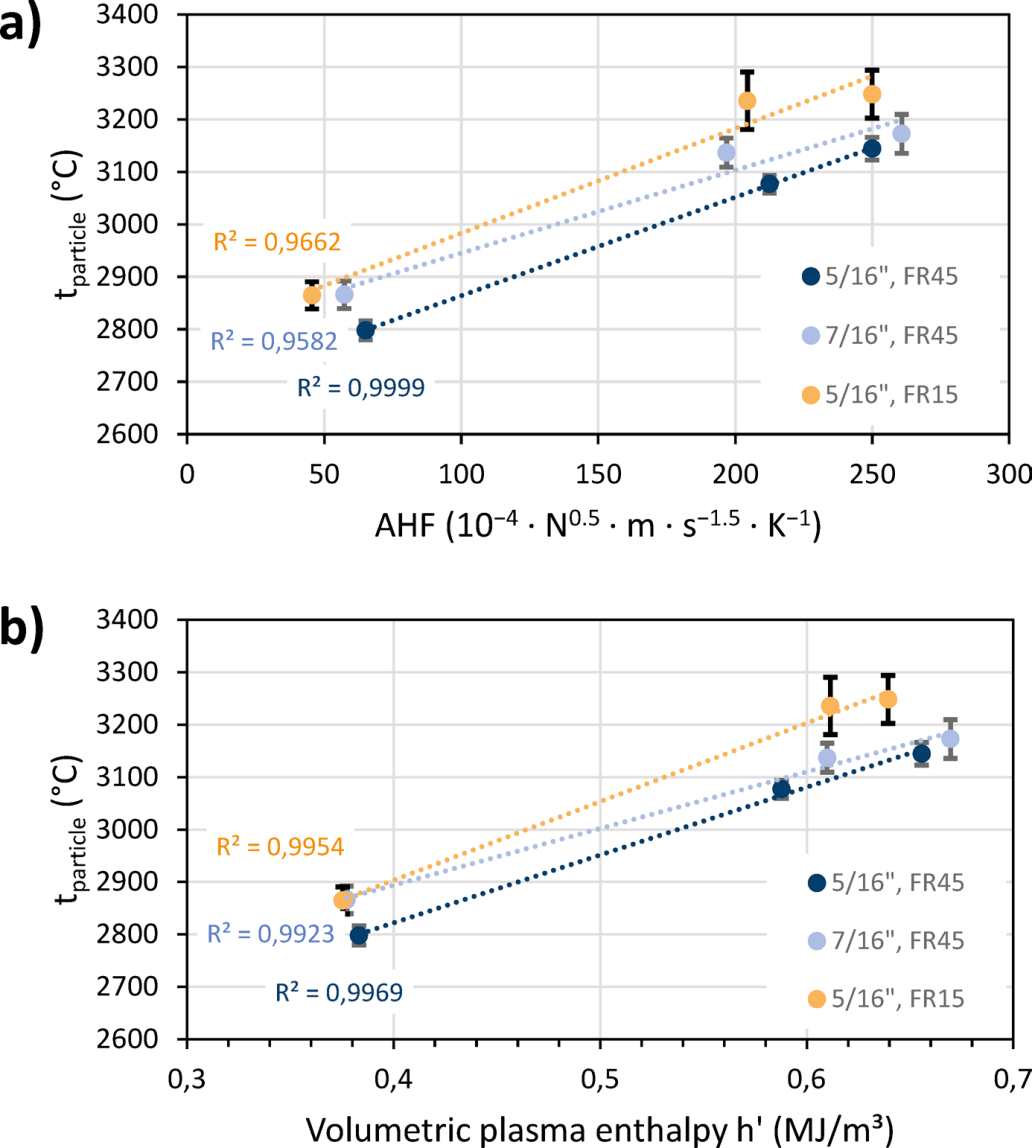
Average measured in-flight temperatures of the particles for the three plasma gas compositions, each for the 7.9 mm (5/16″) and the 11.1 mm (7/16″) nozzles, (**a**) plotted as function of the AAF, (**b**) plotted as function of the volumetric enthalpy at 100 kPa; the FR labels indicate the suspension feed rates of 15 ml/min and 45 ml/min, and the error bars show the temporal variation of the measured temperatures.

Figure 11a shows the average measured in-flight temperatures of the particles for the three plasma gas compositions, each for the 7.9 mm (5/16″) and the 11.1 mm (7/16″) nozzles. The average temporal variation of the measured velocities was found to be ± 1.0%, which is smaller than the 3% measurement accuracy. Two suspension feed rates were set for the smaller nozzle: 15 ml/min and 45 ml/min. The particle temperatures were plotted as a function of the AHF’, which was calculated from the Table 3 data. The thermal conductivity *λ* and the viscosity *η* describe the conditions in the immediate vicinity of the nozzle exit, where the interaction between the plasma gas and the particles predominantly occurs.

A linear correlation is evident indicating that the thermal conductivity *λ* and the dynamic viscosity *η* of the plasma gas are key parameters for particle heating. At the prevailing temperatures, thermal conductivity has a significantly greater effect than viscosity. The highest particle temperatures were obtained at the highest AHF’ values with the N-based parameter closely followed by the Ar-N parameter (see Table 1). It can be concluded that effective heating of the feedstock is promoted by nitrogen’s transport properties in combination with hydrogen. Higher temperatures are consistently achieved with a larger nozzle diameter and a smaller expansion ratio, resulting from a longer particle dwell time in the plasma.

Figure 11b shows the same particle temperature data, but here plotted as a function of the volumetric plasma enthalpy, *h’*. Once again, a linear correlation is evident. Therefore, in addition to the plasma’s transport coefficients, the volumetric enthalpy likewise determines particle heating. This can also be seen in the variation of the suspension feed rate. Apparently, a loading effect occurs when more suspension is exposed to the plasma. Since the amount of energy is limited at a specific enthalpy, lower particle temperatures are achieved. However, increasing the plasma enthalpy enables achieving the same or higher particle temperatures when using larger suspension feed rates. These findings confirm that suspension plasma spraying is an energy-intensive process and that high-enthalpy plasma torches can effectively process large amounts of feedstocks.

## Conclusions

This paper focuses on the role of nitrogen as a primary plasma gas component for thermal spraying. Three plasma gas compositions were used with the Axial III™ plasma torch for suspension plasma spraying: a ternary argon-based composition, a ternary nitrogen-based composition, and a nitrogen-based composition without argon. First, the reasons for considering volumetric enthalpies rather than mass-related enthalpies in this study are explained. Then, the thermodynamic and transport properties of the plasma gas mixtures were calculated and compared. Based on these calculations, ability of acceleration factors (AAF) and ability of heating factors (AHF) were determined and correlated with the measured in-flight particle velocities and temperatures. Such correlations were also established with volumetric plasma enthalpies.

The calculated AAFs did not differ significantly when using the same nozzle. Plasma gas expansion, expressed by the mass density ratio *ρ*_0_*/ρ*, and the dynamic viscosity *η* of the plasma gas push the particle acceleration. However, for the investigated plasma gas compositions, these two factors act in opposite directions. As the nitrogen content increases, the density ratio increases while the dynamic viscosity decreases. Consequently, there were no significant variations in the AAFs, and thus, the measured particle velocities varied indifferently. Dependence on volumetric plasma enthalpy was similarly undifferentiated.

Nitrogen is known for being a high-enthalpy gas. In fact, calculated enthalpies over a wide range of temperatures are higher the higher the nitrogen content. The same is true for hydrogen, but to a lesser extent, due to its lower dissociation enthalpy. A high enthalpy means that more energy is required to bring the plasma gas to a given temperature. Although dissociation enthalpies are released during recombination as the plasma cools, this occurs at much higher temperatures for nitrogen and therefore happens earlier than for hydrogen. Therefore, nitrogen’s relatively high enthalpy does not necessarily cause more effective heat transfer to the particles. In any case, the driving force for heat transfer from the plasma to the feedstock is the temperature difference, not the enthalpy.

Under the boundary conditions of this study—equal currents and total gas flows for all three parameters—the plasma temperatures were found to be quite similar. This result stems from the interaction between the specific enthalpy of the plasma gas composition and the input power in constant current mode, as well as the thermal efficiency. However, similar plasma temperatures do not mean that the feedstock particles are heated equally. Plasma gas mixtures with a high nitrogen content have higher thermal conductivity *λ* and lower viscosity *η*, which ensures more efficient heat transfer and a longer dwell time. This is confirmed by the calculated AHFs, which correlate with the measured particle in-flight temperatures. Therefore, nitrogen can be considered a component that facilitates high heat transfer from the plasma to the particles. This concept is also discussed in the works of Vardelle et al.^21^ and Marple^22^ on nitrogen-based plasmas. Neither study mentions enthalpy.

However, when treating solid or liquid feedstocks at high feed rates requires a lot of energy, the enthalpy of the plasma gas becomes essential. This was demonstrated by varying the suspension feed rate. A distinct loading effect, i.e., lower particle temperatures, was observed at higher feed rates when the plasma enthalpy was kept constant. Nevertheless, an increase in temperature can be achieved by using nitrogen-based plasma gas mixtures, as a correlation was found with the volumetric plasma enthalpy. Therefore, it is evident that suspension plasma spraying is an energy-intensive process.

## Data Availability

The datasets generated and analyzed during the current study are available from the corresponding author on reasonable request.
